# The impact of academic achievement anxiety on perceived stress and menstrual health outcomes among female university students

**DOI:** 10.1186/s12889-026-28553-0

**Published:** 2026-07-21

**Authors:** Nazire Bahar, Meltem Kaydırak, Merve Civelek, İclal Karakaya

**Affiliations:** 1 Faculty of Health Sciences, Department of Midwifery, Istanbul Medipol University, Istanbul, Türkiye; 2 Institute of Graduate Studies, Gynecologic Nursing, Istanbul University-Cerrahpasa, Istanbul, Türkiye; 3 Florence Nightingale Faculty of Nursing, Department of Women’s Health and Gynecologic Nursing, Istanbul University-Cerrahpasa, Istanbul, Türkiye

**Keywords:** Academic achievement anxiety, Perceived stress, Menstrual hygiene behaviors, Menstrual health, Menstrual pain

## Abstract

**Introduction:**

Academic achievement anxiety is a common psychosocial stressor among university students and may be associated with menstrual health outcomes. However, limited research has examined the relationships between academic achievement anxiety, perceived stress, menstrual pain, and menstrual hygiene behaviors simultaneously in female university populations.

**Materials and methods:**

This cross-sectional study included 307 female university students in Turkiye (mean age = 20.59 ± 2.02 years). Participants completed online self-report measures assessing academic achievement anxiety, perceived stress, menstrual pain severity and menstrual hygiene behaviors. Descriptive statistics, Pearson correlations and regression analyses were conducted to examine associations among the variables.

**Results:**

Academic achievement anxiety was positively associated with perceived stress and menstrual pain and negatively associated with genital hygiene behaviors. Multiple linear regression analyses showed that these associations remained significant after controlling for relevant covariates, including age, income level, menstrual regularity, and faculty. Academic achievement anxiety emerged as a significant predictor of perceived stress(β = 0.315, *p* < 0.001), menstrual pain (β = 0.211, *p* < 0.001), and genital hygiene behaviors (β = −0.126, *p* = 0.026).

**Conclusion:**

Academic achievement anxiety was associated with higher perceived stress, increased menstrual pain and slightly less optimal menstrual hygiene practices among female university students. However, these associations were modest, suggesting that academic-related stress may represent one of several factors related to menstrual health outcomes. Addressing academic-related stress within a broader context that includes psychological and structural factors may support student wellbeing. Future research should further explore these relationships using more comprehensive designs.

## Introduction

Approximately 1.8 billion women worldwide experience regular menstrual cycles [1]. Menstrual health is a holistic concept encompassing physical, psychological, and social well-being throughout the menstrual cycle and has increasingly been recognized as a critical public health priority [2] The maintenance of menstrual health is influenced not only by biological processes but also by psychosocial, environmental, and lifestyle-related factors, including stress [3]. In this context, individuals’ stress levels and psychosocial burdens play a significant role in shaping health-related behaviors [4].

Female students in higher education are exposed to a multidimensional stress environment driven by academic performance expectations, as well as biological, psychological, and social determinants identified in previous studies [3, 5]. For adolescents, menstrual health challenges are often linked to pubertal development and the immaturity of the hypothalamic–pituitary–ovarian axis, which can result in irregular cycles and heightened vulnerability to discomfort [6]. In contrast, working women frequently encounter occupational stressors such as workload, shift schedules, and organizational demands, which may interact with menstrual health in ways that affect both productivity and well-being [7]. University students, however, face unique pressures related to academic achievement anxiety and exam stress, which can elevate perceived stress levels and negatively influence menstrual health, thereby impacting their educational participation [8].

Female students in higher education are exposed to a multidimensional stress environment driven by academic performance expectations as well as biological, psychological, and social determinants. Evidence suggests that these cumulative stressors can significantly influence health behaviors [3, 5]. Academic achievement anxiety, in particular, may elevate perceived stress levels by intensifying psychological pressure and concerns regarding performance outcomes. Increased perceived stress not only affects mental well-being but may also have implications for menstrual health, which is an essential component of reproductive health. Recent population-based evidence also demonstrates that menstrual-related symptoms are associated with reduced school attendance and learning participation among young individuals, highlighting the academic impact of menstrual discomfort [9].

From a physiological perspective, stress activates the hypothalamic–pituitary–adrenal (HPA) axis, leading to hormonal imbalances that can disrupt the menstrual cycle. Such disruptions may manifest as irregular menstruation, dysmenorrhea, or premenstrual syndrome [10, 11] Furthermore, elevated stress levels may impair individuals’ ability to maintain adequate menstrual hygiene practices. From a behavioral perspective, this effect can be explained by self-regulation theory, which suggests that individuals have limited psychological resources to manage stress and sustain routine health behaviors [12]. High levels of stress may deplete these resources, making it more difficult to maintain consistent hygiene practices. In addition, increased stress may impose a higher cognitive burden, leading individuals to overlook or postpone routine health behaviors that are not perceived as immediately urgent. Poor menstrual hygiene practices may increase the risk of infections, thereby posing broader public health concerns. Additionally, limited access to menstrual hygiene products has been shown to increase menstruation-related stress, embarrassment, and absenteeism among students, indicating that menstrual hygiene challenges can further elevate psychosocial burden [13].

It should be acknowledged that the relationship between stress and menstrual health may be bidirectional. While menstrual health problems may increase stress levels, stress—particularly academic achievement anxiety—can also influence health behaviors and symptom perception [3, 11]. In the present study, the proposed direction was guided by the focus on examining interrelationships among these variables and evaluating the extent to which academic achievement anxiety contributes to perceived stress and menstrual hygiene behaviors.

Although previous studies have examined the relationship between academic anxiety and stress, as well as the impact of stress on menstrual health separately [10, 11, 14], integrated models addressing these variables simultaneously remain limited. In particular, the associations among academic achievement anxiety, perceived stress, and menstrual hygiene behavior have not been sufficiently explored. This gap highlights an important public health concern, as these factors may influence young women’s health behaviors and long-term reproductive health outcomes.

Therefore, this study aimed to examine the relationships among academic achievement anxiety, perceived stress, and menstrual hygiene behavior and to evaluate the contribution of perceived stress and menstrual hygiene behavior to academic achievement anxiety among female university students.

Research questions;

- (1) What are the mean levels of academic achievement anxiety, perceived stress, and menstrual hygiene behavior among female university students?
- (2) Are there significant relationships between academic achievement anxiety and perceived stress, and between perceived stress and menstrual hygiene behavior?
- (3) Is academic achievement anxiety associated with perceived stress, menstrual hygiene behaviors, and menstrual pain after controlling for relevant covariates?

## Materials and methods

### Study design and setting

A descriptive and correlational research design was used in this study, which was conducted with female university students studying in Turkiye. Data collection took place between 15 March 2025 and 25 June 2025. According to the Higher Education Council [15], approximately 3.685.522 female university students are enrolled in Turkish universities. The aim of this study was to investigate the associations between academic achievement anxiety, perceived stress, menstrual pain, and menstrual hygiene behaviors among female university students. As no prior research has specifically addressed this topic in Turkey, this study seeks to fill an important gap in the literature.

### Sample and criteria for participants

An a priori power analysis was conducted using G*Power software (version X.X) based on a multiple linear regression model (F-test: Linear multiple regression, fixed model, R² deviation from zero). Assuming a medium effect size (f² = 0.25), a significance level of 0.05, a statistical power of 80%, and 10 predictors, the minimum required sample size was calculated as 269 participants. The number of predictors reflected the maximum number of variables anticipated for inclusion in the planned regression analyses during the study design phase. The final sample consisted of 307 female university students who met the inclusion criteria and voluntarily agreed to participate in the study.

Participants were recruited using a convenience sampling approach through online distribution of the survey to female university students studying at different universities in Turkiye. Before completing the questionnaire, participants were screened for eligibility through self-reported questions regarding age, university enrollment status, menstrual status, and psychiatric diagnosis. Eligibility criteria included being 18 years of age or older, currently enrolled in a university, and providing informed consent to participate. Students with amenorrhea due to any cause (e.g., pregnancy or menopause) and those with a diagnosed psychiatric disorder were excluded from the study. Although the study did not aim to obtain a statistically representative sample of all female university students in Turkiye, efforts were made to recruit participants from different academic disciplines to enhance sample diversity.

### Data collection method

The data were collected via an online questionnaire developed by the researchers via the Google Forms platform. The questionnaire was distributed to volunteers via various social media platforms (e.g., Instagram, Facebook, X). Before accessing the survey, the participants were informed in detail about the purpose and scope of the study as well as the voluntary nature of participation. Electronic informed consent was obtained from all participants before participation in the study. The participants could not proceed with the survey questions without confirming the statement “I am voluntarily participating in the study”. The questionnaire was structured so that all questions had to be answered and was designed to be completed in approximately 15 min. This method aims to improve both participant experience and data integrity [16].

### Data collection tools

In this study, demographic questionnaire, the Genital Hygiene Scale (GHBS), the Visual Analog Scale (VAS) and the Perceived Stress Scale (PSS) were used to collect data.

#### Demographic questionnaire

This researcher-developed questionnaire was prepared based on a review of the relevant literature [17, 18] and consisted of 20 items. It was designed to collect information on participants’ sociodemographic, academic, health-related, and menstrual characteristics, including age, year of study, marital status, medical history, age at menarche, menstrual regularity, duration of menstruation, menstrual pain, difficulties attending school during menstruation, and access to menstrual hygiene products. The questionnaire content was developed to ensure alignment with the study objectives and variables of interest.

The Genital Hygiene Behavior Scale (GHBS) was developed by Karahan to assess women’s genital hygiene behaviors. It consists of 23 items on a five-point Likert scale and includes three subscales: general hygiene behavior (GHB), menstrual hygiene (MH) and awareness of normal findings (ANF). The total score ranges from 23 to 115, with higher scores indicating healthier hygiene behaviors. In the original validation study, the Cronbach’s alpha coefficients were reported as 0.80 for the total GHBS, 0.70 for the GHB subscale, 0.74 for the MH subscale, and 0.81 for the ANF subscale [15]. In the present study, the Cronbach’s alpha coefficients were 0.82 for the total GHBS, 0.67 for GHB, 0.66 for MH, and 0.72 for ANF. Although the reliability coefficients of the GHB and MH subscales were slightly below the conventional threshold of 0.70, they were considered acceptable for exploratory research and were therefore retained in the analyses.

The Visual Analog Scale (VAS) is a psychometric instrument consisting of a 10-cm horizontal line used to assess subjective experiences and perceptions. Participants indicate their perceived level of a given construct on a continuum ranging from “none” to “very severe.” In the present study, the VAS was used to assess academic achievement anxiety and menstrual pain [19] To assess academic achievement anxiety, participants were asked, “How anxious do you currently feel about your academic achievement?” and were instructed to rate their anxiety on a scale ranging from 0 (no anxiety) to 10 (extreme anxiety).

The Perceived Stress Scale (PSS), originally developed by Cohen et al. (1983) and adapted into Turkish by Yerlikaya and İnanç (2007), consists of 14 items designed to assess the degree to which individuals perceive their lives as stressful during the previous month. Items 4, 5, 7, and 8 are reverse scored. Total scores range from 0 to 56, with higher scores indicating greater perceived stress. The PSS has demonstrated satisfactory psychometric properties across diverse populations. In the present study, the Cronbach’s alpha coefficient for the scale was 0.72 [17].

### Data cleaning and reliability check

Prior to analysis, the dataset was screened to ensure data quality and response validity. Responses completed in an implausibly short time (e.g., 2–3 min) were excluded, as were cases exhibiting patterns indicative of careless responding, such as straight-line responding (i.e., selecting the same response option across all items), internally inconsistent response patterns, and other indicators of poor response quality. These procedures were implemented to enhance the validity and reliability of the dataset and are consistent with recommended practices for online survey research [12].

In addition, the internal consistency of all study measures was assessed using Cronbach’s alpha coefficients. The reliability estimates indicated acceptable internal consistency for the scales used in the present study.

### Ethical considerations

Ethical approval for this study was obtained from the Istanbul Medipol University Non-Interventional Research Ethics Committee (Approval No: E-10840098-202.3.02-1940/273; Date: March 6, 2025). The study was conducted in accordance with the ethical principles of the Declaration of Helsinki. Informed consent to participate was obtained electronically from all participants prior to their inclusion in the study, and participation was entirely voluntary.

### Data analysis

In this study, statistical analyses were performed using the Statistical Package for the Social Sciences (IBM SPSS) version 22.0. Prior to the main analyses, data were assessed for normality by examining skewness and kurtosis values. All variables met the acceptable criteria for a normal distribution (± 1.5), supporting the use of parametric statistical tests. Descriptive statistics, including frequencies (percentages), means, and standard deviations, were calculated to summarize participants’ sociodemographic characteristics and study variables. Pearson’s correlation analyses were conducted to examine the relationships among academic achievement anxiety, perceived stress, menstrual pain, and menstrual hygiene behaviors. In addition, simple linear regression analyses were performed to assess the associations between academic achievement anxiety and perceived stress, menstrual hygiene behaviors, and menstrual pain.

To address the study research questions, three multiple linear regression models were developed based on the study’s conceptual framework. In each model, academic achievement anxiety was entered as the primary independent variable. Perceived stress, menstrual pain, and genital hygiene behaviors were analyzed as dependent variables in separate models. Relevant covariates were selected based on theoretical considerations and previous literature. Specifically, age and income level were included in the perceived stress model; age and menstrual regularity were included in the menstrual pain model; and age, income level, and faculty type were included in the genital hygiene behavior model. Statistical significance was set at *p* < 0.05.

## Results

The sociodemographic and menstrual characteristics of the participants are presented in Table 1. The mean age of the students was 20.59 ± 2.02 years, and the mean age at menarche was 12.90 ± 1.47 years. Descriptive statistics for the study variables are presented in Table 2. Participants had a mean Academic Achievement Anxiety score of 6.35 ± 2.47 and a mean Perceived Stress Scale score of 29.94 ± 6.88, and Menstrual Pain Scale score of 5.95 ± 2.74. The correlations among the study variables are presented in Table 2. Academic achievement anxiety was positively correlated with perceived stress (*r* = 0.32, *p* < 0.01) and menstrual pain (*r* = 0.21, *p* < 0.01), while weak negative correlations were observed with genital hygiene behaviors (*r* = − 0.13, *p* < 0.05) and general hygiene behaviors (*r* = − 0.16, *p* < 0.05). Similarly, perceived stress was negatively correlated with genital hygiene behaviors (*r* = − 0.12, *p* < 0.05) and general hygiene behaviors (*r* = − 0.15, *p* < 0.05) and positively correlated with menstrual pain (*r* = 0.15, *p* < 0.01).

**Table 1:** Information on the socio-demographic and menstrual characteristics of the female students (*N* = 307)

Variable	Mean ± SD	Min – max	Variable	*n*	%
Age (min- max)	20.59 ± 2.02	18–30	**Regularity of menstruation**		
Age at menarche	12.90 ± 1.47	10–19	Regular	183	59.6
Duration of menstruation (days)	5.79 ± 1.46	3–15	Irregular	38	12.4
Number of pads used per day	4.71 ± 3.79	0–10	Partly regular	86	28.0
Family type	**N**	**%**	**Type of menstrual hygiene product used**		
Nuclear	240	78.2	Sanitary Pad	304	99.0
Extended	51	16.6	Cloth Pad	8	2.6
Broken	16	5.2	Tampon	9	2.9
Income level			**Ability to buy menstrual products**		
Low	66	21.5	Easy	269	87.6
Medium	191	62.2	Difficult	38	12.4
High	50	16.3	**Difficulty attending school during menstruation**		
Place of residence			Always	58	18.9
With family	194	63.2	Sometimes	197	64.2
Dormitory, own house	113	36.8	Never	52	16.9
Employment status			**Faculty**		
Working	48	15.6	Health Sciences*	167	54.4
Not Working	259	84.4	Other Disciplines **	140	45.6

*Health sciences: students enrolled in degree programmes in medicine, dentistry, pharmacy, nursing and midwifery

**Other disciplines: students enrolled in degree programmes in the fields of education, engineering, law, communication, science and literature, art and design and social sciences

**Table 2:** Correlation between academic achievement anxiety, perceived stress, menstrual hygiene behaviour and menstrual pain and the total scale scores (N = 307)

	PSS	GHBS	GHB	MH	ANF	Menstrual pain
Academic Achievement Anxiety Level, VAS	0.32**	-0.13^*^	-0.16^**^	-0.04	-0.10	0.21**
Perceived Stress Scale	1	-0.12^*^	-0.15^**^	-0.04	0.08	0.15^**^

Scales and Subscale	Mean ± SD			Min – Max		
Academic Achievement Anxiety Level, VAS	6.35 ± 2.47			0 – 10		
Perceived Stress Scale	29.94 ± 6.88			3 – 53		
Genital Hygiene Behaviors Scale	93.87 ± 9.92			56 - 113		
General Hygiene Behaviors Subscale	48.95 ± 5.58			26 - 60		
Menstrual Hygiene Subscale	33.12 ± 4.48			19 - 40		
Awareness of Normal Findings Subscale	11.79 ± 2.38			3- 15		
Menstrual pain, VAS	5.95±2.74			0-10		

*Correlation is significant at the 0.05 level (2-tailed)

**Correlation is significant at the 0.01 level (2-tailed); VAS: Visual Analogue Scale

As presented in Table 3, simple linear regression analyses showed that academic achievement anxiety was a significant positive predictor of perceived stress (B = 0.891, β = 0.320, t = 5.899, *p* < 0.001) and menstrual pain (B = 0.238, β = 0.214, t = 3.831, *p* < 0.001). Conversely, academic achievement anxiety was negatively associated with genital hygiene behaviors (B = − 0.547, β = −0.136, t = − 2.405, *p* = 0.017) and the hygiene behaviors subdimension (B = − 0.364, β = −0.161, t = − 2.849, *p* = 0.005).

**Table 3:** Simple linear regression analyses with academic achievement anxiety as the independent variable

Dependent variable	Independent variable	B	Std. Error	β (Beta)	t	*p*	*R*²
PSS	**Academic achievement Anxiety**	0.891	0.151	0.320	5.899	***< 0.001***	0.102
GHBS Total Score		-0.547	0.228	-0.136	-2.405	*0.017*	0.019
GHB Subscale		-0.364	0.128	-0.161	-2.849	***0.005***	0.026
Menstrual Pain (VAS)		0.238	0.062	0.214	3.831	***< 0.001***	0.046

*VAS* Visual Analogue Scale, *PSS* Perceived Stress Scale, *GHBS* Genital Hygiene Behaviors Scale, *GHB* General Hygiene Behaviors, *MH* Menstrual Hygiene, *ANF* Awareness of Normal Findings

To further examine whether academic achievement anxiety was associated with menstrual health outcomes after controlling for relevant covariates, three multiple linear regression analyses were conducted (Table 4). The model predicting perceived stress was statistically significant (F(4,302) = 9.164, *p* < 0.001), explaining 10.8% of the variance. Academic achievement anxiety was positively associated with perceived stress (β = 0.315, *p* < 0.001), whereas age and income level were not significant predictors. The model predicting menstrual pain was also statistically significant (F(4,302) = 3.823, *p* = 0.005), accounting for 4.8% of the variance. Academic achievement anxiety remained a significant positive predictor of menstrual pain (β = 0.211, *p* < 0.001), while age and menstrual regularity were not significantly associated with menstrual pain. The model predicting genital hygiene behaviors was statistically significant (F(5,301) = 4.298, *p* = 0.001), explaining 6.7% of the variance. Academic achievement anxiety was negatively associated with genital hygiene behaviors (β = −0.126, *p* = 0.026). In contrast, age (β = 0.172, *p* = 0.003) and high income level (β = 0.175, *p* = 0.011) were positively associated with genital hygiene behaviors. Faculty type and moderate income level were not significant predictors.

**Table 4:** Multiple linear regression models predicting perceived stress, menstrual pain, and genital hygiene behaviors

Multiple Linear Regression Analysis Predicting Perceived Stress						
Predictor	B	SE	β	t	*p*	95% CI for B
Constant	27.690	4.230	–	6.546	< 0.001	19.366–36.014
Academic Achievement Anxiety	0.877	0.152	0.315	5.775	< 0.001	0.578–1.176
Age	-0.114	0.188	-0.034	-0.609	0.543	-0.485–0.256
Moderate vs. Low Income	-1.158	0.951	-0.082	-1.218	0.224	-3.028–0.713
High vs. Low Income	-1.454	1.236	-0.078	-1.176	0.240	-3.887–0.978
*Model statistics*: R² = 0.108, Adjusted R² = 0.096, F(4,302) = 9.164, *p* < 0.001						
Multiple Linear Regression Analysis Predicting Menstrual Pain						
Constant	5.657	1.636	—	3.459	0.001	2.439, 8.876
Academic achievement anxiety	0.234	0.063	0.211	3.731	< 0.001	0.111, 0.358
Age	-0.059	0.076	-0.043	-0.774	0.440	-0.209, 0.091
Irregular vs. Regular menstruation	-0.060	0.480	-0.007	-0.126	0.900	-1.006, 0.885
Partly regular vs. Regular menstruation	0.101	0.355	0.017	0.283	0.777	-0.598, 0.799
Model statistics: R² = 0.048, Adjusted R² = 0.036, F(4,302) = 3.823, *p* = 0.005						
Multiple Linear Regression Analysis Predicting Genital Hygiene Behaviors						
Constant	78.185	6.279	–	12.451	< 0.001	65.828, 90.542
Academic Achievement Anxiety	-0.504	0.226	-0.126	-2.232	0.026	-0.948, -0.060
Age	0.845	0.278	0.172	3.041	0.003	0.298, 1.392
Moderate vs. Low Income	1.713	1.404	0.084	1.220	0.223	-1.050, 4.476
High vs. Low Income	4.687	1.828	0.175	2.563	0.011	1.089, 8.285
Other Disciplines vs. Health Sciences	-0.763	1.119	-0.038	-0.682	0.496	-2.964, 1.438
Model statistics: R² = 0.067, Adjusted R² = 0.051, F(5,301) = 4.298, *p* = 0.001						

*B* unstandardized regression coefficient, *SE* standard error, *β* standardized regression coefficient, *CI* confidence interval. Income level was entered using dummy variables with low income as the reference category. Menstrual regularity was entered using dummy variables with regular menstruation as the reference category. Faculty was entered using dummy variables with Health Sciences as the reference category

## Discussion

This study examined the associations between academic achievement anxiety, perceived stress, menstrual pain, and menstrual hygiene behaviors among female university students. Overall, the findings suggest that academic achievement anxiety may be associated with higher perceived stress and increased menstrual pain, while showing a weak association with menstrual hygiene behaviors. These results indicate that academic-related psychological burden may be linked to both psychological and physical aspects of menstrual health; however, the observed associations are modest in magnitude and should be interpreted with caution.

Consistent with previous literature, these findings support the notion that academic-related stressors may contribute to psychological distress and menstrual-related symptoms among university students. Prior research has demonstrated that stress can influence menstrual health through physiological pathways, including dysregulation of the hypothalamic–pituitary–adrenal axis and subsequent hormonal alterations, as well as through behavioral mechanisms affecting self-regulation and health practices [3, 10–12]. Nevertheless, due to the cross-sectional design of the present study, causal interpretations cannot be made.

The results also suggest that menstrual health-related challenges, including menstrual pain and hygiene management, remain relevant concerns in university populations. Previous studies have similarly reported that menstrual symptoms are common among students and may be influenced by stress and lifestyle factors [20–22]. In addition, difficulties in accessing menstrual hygiene products have been identified as a structural barrier affecting both menstrual health management and academic participation [23–26]. These findings highlight the importance of considering menstrual health within broader structural and institutional contexts.

Menstrual hygiene behavior scores in this study were comparable to those reported in previous university-based studies [17, 27, 28]. suggesting relative consistency across student populations with diverse academic backgrounds. This may indicate that menstrual hygiene behaviors are influenced more by broader socio-cultural and structural factors than by academic discipline alone, underscoring the importance of university-wide menstrual health education initiatives.

The findings further suggest that academic achievement anxiety may be associated with increased perceived stress and menstrual pain. This pattern is consistent with previous research indicating that academic-related stressors may contribute to both psychological strain and menstrual symptoms [18, 29–31]. From a theoretical and physiological perspective, stress-related mechanisms, including hormonal responses and challenges in self-regulation, may help explain these relationships [12]. However, these associations should be interpreted with caution due to the cross-sectional design of the study.

In contrast, the association between academic achievement anxiety and menstrual hygiene behaviors was relatively weak. This limited association may, in part, be explained by the relatively high level of hygiene practices observed in the study sample. The mean GHBS score (93.87 out of 115) indicates that participants generally reported relatively high levels of genital hygiene behaviors, which may have resulted in restricted variability and attenuated the observed associations. In addition, menstrual hygiene behaviors are typically routine and well-established practices. From a self-regulation perspective, such habitual behaviors become increasingly automatic over time and therefore require fewer self-regulatory resources than novel or more complex behaviors. Consequently, they may be relatively resistant to stress-related cognitive depletion, which could partly explain the weak association observed between academic achievement anxiety and menstrual hygiene behaviors. Nevertheless, menstrual hygiene practices are also likely influenced by a broader set of factors, including socioeconomic conditions, access to hygiene products, cultural norms, and individual awareness [13, 31–34]. Accordingly, although academic achievement anxiety was significantly associated with menstrual hygiene behaviors, perceived stress, and menstrual pain, the observed effect sizes were small, suggesting that academic achievement anxiety represents only one of several factors contributing to menstrual health outcomes rather than a primary determinant. Therefore, these findings should be interpreted with caution to avoid overstating the practical significance of the observed associations. From a methodological perspective, the present study used a convenience sampling strategy via social media platforms, which may limit the generalizability of the findings due to potential self-selection bias and overrepresentation of digitally active students [8, 16]. This limitation should be considered when interpreting the results.

Additionally, participants with diagnosed psychiatric disorders were excluded to reduce potential confounding effects related to clinically significant mental health conditions. However, this decision may also limit variability in anxiety-related constructs and should be considered when interpreting the findings [3, 11, 14].

The regression model presented in Table 4 examined reverse directional associations, testing whether menstrual outcomes and perceived stress collectively predicted academic achievement anxiety. This analysis was included as an exploratory approach to examine potential bidirectional relationships. However, the cross-sectional design does not allow conclusions regarding directionality, and these results should be interpreted cautiously [11, 18]. 

Finally, scatterplot inspection was conducted to evaluate potential non-linear relationships between variables, including stress and menstrual hygiene behaviors. No clear evidence of non-linear patterns was observed; therefore, linear models were retained for analysis. Nonetheless, future studies may benefit from more advanced modeling approaches to further explore potential non-linear associations [12, 35].

Taken together, these findings suggest that academic achievement anxiety may be one of several interrelated factors associated with menstrual health outcomes among female university students. However, the relatively small effect sizes highlight the need for cautious interpretation and suggest that menstrual health is shaped by a broader constellation of psychological, behavioral, and structural factors [3, 10, 11].

From a practical perspective, these findings suggest that interventions aimed at supporting stress management among university students may have potential benefits for menstrual health. Previous studies have emphasized the role of stress-related factors in menstrual symptoms and young women’s wellbeing [10, 36, 37]. In addition, improving access to menstrual hygiene products and strengthening menstrual health awareness at institutional levels may help support students’ health and academic participation [31–34]. However, given the modest effect sizes observed, such interventions should be considered as part of broader, multi-level strategies addressing both individual and structural determinants.

Future research should employ longitudinal designs to clarify causal pathways and directionality among these variables. In addition, incorporating multidimensional measures of academic anxiety and contextual variables such as socioeconomic status, health literacy, and access to menstrual hygiene resources may provide a more comprehensive understanding of menstrual health in university populations [12, 13].

## Conclusion

This study suggests that academic achievement anxiety may be associated with perceived stress, menstrual pain, and menstrual hygiene behaviors among female university students. However, these relationships appear to be modest, indicating that academic anxiety represents only one of several factors influencing menstrual health.

These findings highlight the potential importance of addressing academic-related stress within a broader context that includes psychological, behavioral, and structural factors. Future research should further explore these relationships using more comprehensive designs and variables.

### Limitations

Several limitations should be considered when interpreting the findings. First, academic achievement anxiety was assessed using a single-item VAS, which may not fully capture the multidimensional nature of academic anxiety. Second, the internal consistency coefficients of two GHBS subscales were slightly below the recommended threshold of 0.70; therefore, findings related to these subscales should be interpreted with caution. Third, the phase of the menstrual cycle at the time of survey completion was not assessed or controlled for in the analyses. As menstrual symptoms and related experiences may vary across the menstrual cycle, this factor may have influenced participants’ responses. Furthermore, participants generally demonstrated high GHBS scores, resulting in relatively restricted variability in genital hygiene behaviors. This limited variability may have attenuated the observed associations and should be considered when interpreting the magnitude of the relationships.

Participants were recruited through a voluntary online survey using a convenience sampling approach. Consequently, selection bias cannot be ruled out, as students experiencing menstrual symptoms or concerns related to menstrual health may have been more likely to participate than those without such experiences. Therefore, the findings may not be fully representative of all female university students. Finally, the cross-sectional design precludes causal inferences and limits conclusions regarding the directionality of the observed associations.

## Data Availability

The data that support the findings of this study are available from the corresponding author upon reasonable request.

## Abbreviations

HPA: Hypothalamic–Pituitary–Adrenal
GHBS: Genital Hygiene Scale
VAS: Visual Analog Scale
PSS: Perceived Stress Scale
GHB: General Hygiene Behavior
MH: Menstrual Hygiene
ANF: Awareness Of Normal Findings
IBM SPSS: Statistical Package for the Social Sciences

## References

[1] World Health Organization (WHO). WHO statement on menstrüel health and rights. 2022. Available from: https://www.who.int/news/item/22-06-2022-who-statement-on-menstrüel-health-and-rights. [Cited 2025 May 15].

[2] Kansiime C, Hytti L, Nelson KA, Torondel B, Francis SC, Tanton C, et al. Menstrual health interventions, schooling, and mental health problems among Ugandan students (MENISCUS): study protocol for a school-based cluster-randomised trial. Trials. 2022;23(1):759. 10.1186/s13063-022-06672-4. PMC9449307 36071530

[3] Alshdaifat E, Absy N, Sindiani A, AlOsta N, Hijazi H, Amarin Z, et al. Premenstrual Syndrome and Its Association with Perceived Stress: The Experience of Medical Students in Jordan. Int J Womens Health. 2022;14:777–85. 10.2147/IJWH.S361964. PMC9206437 35726296

[4] Misra R, McKean M. College students academic stress and its relation to their anxiety, time management, and leisure satisfaction. Am J Health Stud. 2000;16:41–51.

[5] Karaman MA, Lerma E, Vela JC, Watson JC. Predictors of academic stress among college students. J Coll Couns. 2019;22(1):41–55. 10.1002/jocc.12113.

[6] Saei Ghare Naz M, Farahmand M, Dashti S, Ramezani Tehrani F. Factors Affecting Menstrual Cycle Developmental Trajectory in Adolescents: A Narrative Review. Int J Endocrinol Metab. 2022;20(1). 10.5812/ijem.120438. PMC8994833 35432553

[7] Ortynsky M, Cloutier A, Byrne A, Carleton EL. Ebbs and flows: A within-person study of menstruation and work performance. J Occup Health Psychol. 2025;30(3):136–55. 10.1037/ocp0000404. 40455499

[8] Munro AK, Hunter EC, Hossain SZ, Keep M. A systematic review of the menstrual experiences of university students and the impacts on their education: A global perspective. PLoS ONE. 2021;16(9):e0257333. 10.1371/journal.pone.0257333. PMC8432759 34506544

[9] Abrahamsson K, Åkerman E, Ström S, Söderman L, Källberg H, Klingberg-Allvin M. Menstrual-related symptoms and absence from school among young people in Sweden: a stratified, randomized, population-based survey. BMC Public Health. 2025;25(1). 10.1186/s12889-025-24705-w. PMC12553222 41136930

[10] Yaşar Ö. The effect of perceived stress on menstrual complaints in university students. Women Health. 2024;64(4):341–9. 10.1080/03630242.2024.2337705. 38556786

[11] Rafique N, Al-Sheikh MH. Prevalence of menstrual problems and their association with psychological stress in young female students studying health sciences. Saudi Med J. 2018;39(1):67–73. 10.15537/smj.2018.1.21438. PMC5885123 29332111

[12] Dorina I, Mullan B, Boyes M, Liddelow C. Utility of temporal self-regulation theory in health and social behaviours: A meta‐analysis. Br J Health Psychol. 2023;28(2):397–438. 10.1111/bjhp.12631. 36404715

[13] Hammad T, Alsaffar M, Taosin Ashin N, Rashid K, Gangat A, Sreedharan J. Menstrual hygiene perceptions and need for free sanitary products: a cross-sectional study in a UAE university setting. BMC Public Health. 2025;25(1). 10.1186/s12889-025-23929-0. PMC12465756 40999381

[14] Kahal F, Alshayeb S, Torbey A, Al Helwani O, Kadri S, Helwani A, et al. The prevalence of menstrual disorders and their association with psychological stress in Syrian students enrolled at health-related schools: A cross‐sectional study. Int J Gynecol Obstet. 2024;164(3):1086–93. 10.1002/ijgo.15152. 37743817

[15] Council of Higher Education. Higher Education Statistics. Number of students in higher education institutions by level of study, 2023–2024 academic year. 2024. Available from: https://istatistik.yok.gov.tr/. [Cited 2025 Feb 5].

[16] Curran PG. Methods for the detection of carelessly invalid responses in survey data. J Exp Soc Psychol. 2016;66:4–19. 10.1016/j.jesp.2015.07.006.

[17] Yağız Altıntaş R, Bakır S, Gül İ, Kavlak O. The effect of nursing students’ attitudes toward menstruation on genital hygiene behaviors. Turkish J Family Med Prim Care. 2021;15(3):568–75. 10.21763/tjfmpc.931262.

[18] Novelia S, Syamsiah S, Wenola W. The Relationship of Stress Levels and Menstrual Cycle among Female Students. Health Technol J (HTechJ). 2023;1(4):381–5. 10.53713/htechj.v1i4.44.

[19] Wewers ME, Lowe NK. A critical review of visual analogue scales in the measurement of clinical phenomena. Res Nurs Health. 1990;13(4):227–36. 10.1002/nur.4770130405. 2197679

[20] Demeke E, Zeru AB, Tesfahun E, Mohammed WB. Effect of menstrual irregularity on academic performance of undergraduate students of Debre Berhan University: A comparative cross sectional study. PLoS ONE. 2023;18(1):e0280356. 10.1371/journal.pone.0280356. PMC9879450 36701333

[21] Alhammadi MH, Albogmi AM, Alzahrani MK, Shalabi BH, Fatta FA, AlBasri SF. Menstrual cycle irregularity during examination among female medical students at King Abdulaziz University, Jeddah, Saudi Arabia. BMC Womens Health. 2022;22(1):367. 10.1186/s12905-022-01952-2. PMC9446759 36068523

[22] Mittiku YM, Mekonen H, Wogie G, Tizazu MA, Wake GE. Menstrual irregularity and its associated factors among college students in Ethiopia, 2021. Front Glob Womens Health. 2022;3. 10.3389/fgwh.2022.917643. PMC9445616 36081684

[23] Gümüşay M, Acar Z. Nursing students’ menstrual experiences and period poverty. Bingöl Üniversitesi Sağlık Dergisi. 2024;5(3):601–10. 10.58605/bingolsaglik.1550846.

[24] World Health Organization (WHO), United Nations Children’s Fund (UNICEF). Progress on drinking water, sanitation and hygiene in schools 2015–2023: Special focus on menstrüel health. 2024. Available from: https://www.who.int/news/item/28-05-2024-global-report-reveals-major-gaps-in-menstrüel-health-and-hygiene-in-schools. [Cited 2025 Aug 15].

[25] Proff AA, Fatima S, Limón MLS. Becoming women: period. Perceptions of barriers and facilitators to menstrual hygiene management programs for Pakistani girls. Front Public Health. 2023;11. 10.3389/fpubh.2023.1083688. PMC10457679 37637817

[26] Suleman A, Krishna S, Krishnakumar D, Nemoto K, Nguyễn MLT, Mehta SD. A pilot survey of students’ menstrual attitudes, experiences, and needs on an urban university campus. Women’s Health. 2024;20. 10.1177/17455057241254713. PMC11143816 38819035

[27] Tiryaki S, Gezginci E, Göktaş S. Determining the genital hygiene behaviors of health sciences faculty students. İnönü Üniversitesi Sağlık Hizmetleri Meslek Yüksek. Okulu Dergisi. 2021;9(2):510–22. 10.33715/inonusaglik.843789.

[28] Hadımlı A, Akan A, Kardeş G, Akkurt B, Saydam BK. Determination of menstrual hygiene management and genital hygiene behaviors of students: A university example from Turkey. Revista da Escola de Enfermagem da USP. 2024;58. 10.1590/1980-220x-reeusp-2024-0113en. PMC11611317 39621939

[29] Jones LI, Simbaña A, Lozada Velasteguí DA, Pérez Sánchez AS, Harris N, Wiseman N, et al. An exploration into the menstrual health of ecuadorian university students: A mixed-methods study. Women’s Reproductive Health. 2025;1–17. 10.1080/23293691.2025.2524375.

[30] Govind GK, Devi G. Academic stress on menstrual cycle among adolescents. Int J Indian Psychol. 2023;11(3):2635–43. 10.25215/1103.287.

[31] Matsuura Y, Tran NH, Yasui T. Association between menstruation-related symptoms and the type of stress in Japanese female university students: A prospective cohort study from admission to the second year. Women. 2024;4:254–64. 10.3390/women4030019.

[32] Turkish Statistical Institute (TurkStat). Women in Statistics in Türkiye 2024. Turkey. 2024. Available from: https://www.tuik.gov.tr/media/announcements/istatistiklerle_kad%C4%B1n2024.pd. [Cited 2025 Feb 15].

[33] Ulubay M, Fıdan U, Öztürk M. The decreasing age of menarche in Turkey: global warming, socioeconomic development, and environmental factors. 2023.27(14):6780–4. 10.26355/eurrev_202307_33148. 37522688

[34] Karakaş S, Dikici A, Oyardı G. Determining coping methods for premenstrual syndrome among university students. J Health Sci Istanbul Rumeli Univ. 2023;1(2):42–58.

[35] Karaküçük S, Sönmez M, Kenç İ. Determining the frequency of premenstrual syndrome symptoms and coping methods using a PMS diary among university students. J Adnan Menderes Univ Health Sci Fac. 2022;6(3):419–30. 10.46237/amusbfd.1085050.

[36] Erbaş N, Altunbaş N. According to some variables in girls a high school determining the premenstrual syndrome violence and perceped stress level. J Acibadem Univ Health Sci. 2021;12(2). 10.31067/acusaglik.849971.

[37] Naw J, Jacquelyn D. Anjana Sinha. Menstrual pain, depression, anxiety and stress among women. Int J Indian Psychol. 2023. 10.25215/1102.148.

